# Is Semantic Processing During Sentence Reading Autonomous or Controlled? Evidence from the N400 Component in a Dual Task Paradigm

**DOI:** 10.5709/acp-0170-2

**Published:** 2015-06-30

**Authors:** Annette Hohlfeld, Manuel Martín-Loeches, Werner Sommer

**Affiliations:** 1 Center for Human Evolution and Behavior, UCM-ISCIII, Madrid; 2 Department of Psychobiology, Complutense University of Madrid; 3 Department of Psychology, Humboldt-University at Berlin

**Keywords:** N400, dual task, semantic processing, reading, automaticity, P600

## Abstract

The present study contributes to the discussion on the automaticity of semantic processing. Whereas most previous research investigated semantic processing at word level, the present study addressed semantic processing during sentence reading. A dual task paradigm was combined with the recording of event-related brain potentials. Previous research at word level processing reported different patterns of interference with the N400 by additional tasks: attenuation of amplitude or delay of latency. In the present study, we presented Spanish sentences that were semantically correct or contained a semantic violation in a critical word. At different intervals preceding the critical word a tone was presented that required a high-priority choice response. At short intervals/high temporal overlap between the tasks mean amplitude of the N400 was reduced relative to long intervals/low temporal overlap, but there were no shifts of peak latency. We propose that processing at sentence level exerts a protective effect against the additional task. This is in accord with the attentional sensitization model (Kiefer & Martens, 2010), which suggests that semantic processing is an automatic process that can be enhanced by the currently activated task set. The present experimental sentences also induced a P600, which is taken as an index of integrative processing. Additional task effects are comparable to those in the N400 time window and are briefly discussed.

## Introduction

There is an ongoing debate whether semantic processing during language perception is automatic or controlled. The classic view of automaticity (e.g., Posner & Snyder, 1975; Schneider & Shiffrin, 1977) holds that automatic cognitive processes are autonomous and independent of top-down control. There is evidence from both the visual modality (i.e., reading) as well as from the acoustic modality (i.e., hearing) supporting the assumption of automatic processing in the classical sense. The so-called Stroop effect (cf. MacLeod, 1991, for a review) strongly suggests that semantic processing during reading is highly automatic; during naming the color of the ink in which a color word such as *blue* is printed, participants cannot avoid reading the word while accessing the name of the ink color. This is reflected in an increase of naming latencies for incongruent (e.g., *blue* printed in red) compared to congruent trials. The usual interpretation for this finding suggests that during access to a word form, activation automatically spreads to the semantic information encoded by that word. This mechanism does not require attention or awareness (Collins & Loftus, 1975). Similar interpretations are given for results from homophone priming (e.g., Lesch & Pollatsek, 1993).

In contrast, other studies argue for the non-automaticity of semantic processing. For example, in prime-target paradigms semantic priming during reading can be modulated by the task related to the prime (prime task effect, Maxfield, 1997). If the task directs attention to non-semantic properties of the prime, as required in letter search, the usually very robust semantic priming effect is reduced as compared to a semantic task, such as naming. Maxfield suggested that attentional mechanisms, required by the task-related focus of mental resources, affect semantic processing during reading. The fact that semantic processing is influenced by attention was taken as evidence for its non-automatic nature.

In addition to measuring performance the recording of event-related brain potentials (ERPs) is very useful in investigating online-processing of linguistic material. The N400 component of the ERP is generally believed to be a modality-unspecific indicator of semantic processing. This component was first reported in the visual modality by Kutas and Hillyard (1984) as a negative potential, maximal at around 400 ms. It is elicited by words that do not fit the semantic context provided by a preceding sentence fragment. The time course of the N400 indicates the temporal dynamics of semantic processing, with onset and peak latency as time markers of its beginning and maximal activity, respectively (Van Petten, Coulson, Rubin, Plante, & Parks, 1999). Chwilla, Brown, and Hagoort (1995) proposed to interpret the N400 as a reflection of post-lexical integration processes. Alternatively, Kutas and Federmeier (2011) characterized the N400 as indexing the effort of accessing long-term multimodal lexico-semantic memory. Similar to the behavioral experiments reported above, electrophysiological studies using N400 yielded ambivalent results supporting or contradicting the automaticity of semantic processing.

Studies using the attentional blink paradigm (Rolke, Heil, Streb, & Henninghausen, 2001; Vogel, Luck, & Shapiro, 1998) reported N400 components to unattended verbal stimuli, supporting the automaticity of semantic processing. In contrast are findings from Holcomb (1988), who required participants in a lexical decision task to attend to or ignore semantic relationships between words and, moreover, varied the proportion of related prime-target pairs. An enhancement of the N400 was found when participants attended semantic relationships and when related prime-target pairs were relatively frequent. Holcomb interpreted these findings as evidence for the attention dependency of the N400 and its underlying semantic processes. Thus, semantic processing would be subject to top-down control and hence should be considered controlled rather than automatic. Further evidence for the controlled nature of semantic processing comes from studies of McCarthy and Nobre (1993), Bentin, Kutas, and Hillyard (1993) as well as from Chwilla, Brown, and Hagoort (1995), reporting effects of attention on the N400 in various tasks.

In sum, with respect to the automatic or controlled nature of semantic processing there is contradictory evidence from the visual as well as from the acoustic modality with different indicators and paradigms. As discussed by Kiefer and Martens (2010) in terms of the classical view of automaticity hardly any process would qualify to be automatic. In various studies Kiefer and colleagues have shown modulating effects on the N400 during masked priming (Adams & Kiefer, 2012; Kiefer, 2002; Kiefer & Spitzer, 2000; Martens, Ansorge, & Kiefer, 2011). Whereas a semantic induction task enhanced the N400 when it was presented immediately before the masked prime, a perceptual task attenuated the N400 (Kiefer & Martens, 2010). Furthermore, unconscious semantic processing was modulated by a cue, presented immediately before the masked prime (Kiefer & Brendel, 2006). The authors argue that the cue attracts attention to the semantic processing stream, thereby enhancing the N400 relative to conditions with a longer interval between cue and masked prime. Interestingly, also task difficulty of a preceding primary task affected masked priming (Martens & Kiefer, 2009). Masked priming effects were attenuated when participants decided whether a primary word started or ended with a letter of closed or open shape (in contrast to an easier decision task about whether the primary word contained a capital letter). Martens and Kiefer (2009) concluded that unconscious semantic processing depends on attentional resources.

Kiefer and Martens (2010) integrated these findings in their attentional sensitization model of automatic processing. This model suggests that semantic processing has to be considered as an automatic process because it is triggered unconsciously without intention. Nevertheless top-down control is still possible to the extent that semantic processing is influenced by the configuration of the cognitive system, depending on the task at hand. Thus, the cognitive system can increase attentional sensitivity to task-relevant pathways (i.e., attentional sensitization). As a consequence, neural activity devoted to semantic processing can be diminished when a different, non-semantic task set becomes relevant. Such a view integrates the controversial findings on semantic processing cited above and makes them appear less heterogeneous.

Whereas previous studies investigated the automaticity of semantic processing at the level of *word* pairs, there are to the best of our knowledge no studies at sentence level. In contrast to the processing of word pairs, access to semantic information during sentence reading is presumably more complex. During reading of single words lexical candidates have to be identified in the mental lexicon (Dietrich, 2002; Hagoort & Brown, 2000). When a whole sentence is read morpho-syntactic properties as well as lexical constraints have to be taken into consideration in order to create fuller syntactic structures and define thematic roles. Thus, on the one hand, at sentence level task load is high, because of several ongoing cognitive processes. On the other hand, the pathway of semantic processing might be strengthened, for example, due to continuous priming from one word to the next.

In the present study it was of specific interest whether semantic processing at sentence level would be modulated by an additional task and what the pattern of N400-modulation would look like. To this aim a psychological refractory period (PRP) paradigm was applied (cf. Exp. 2). Sentence reading was combined with an additional task requiring the processing of non-linguistic material: a high or low tone had to be classified according to pitch. The temporal overlap of sentence reading and the additional task was manipulated by varying the stimulus onset asynchrony (SOA) between the tone and the target word, allowing for a precise control of the time-course of interference. More specifically, in the paradigm used here, a tone (S1) and a visually presented target word (S2) - the adjective of a sentence - were presented at one of three SOAs (100, 400, or 700 ms). The target word was always the penultimate word in the experimental sentences (cf. Appendix for example sentences). Sentences required acceptability judgments immediately after sentence termination. Semantic processing was assessed by a sentence acceptability task based on appropriate and inappropriate Spanish noun-adjective pairings. Inappropriate adjectives were expected to induce N400 components in the ERP.

From word level studies using PRP paradigms, different interference patterns are known, such as a temporal delay of semantic processing, reflected in a shift of peak latency of the N400 component (Hohlfeld, Sangals, & Sommer, 2004; Lien, Ruthruff, Cornett, Goodin, & Allen, 2008) or N400 amplitude reductions (Hohlfeld & Sommer, 2005). The present study investigated the interference pattern at sentence level. Predictions for the sentence level depend on the model of automaticity held. If we stick to the classical dichotomy of automatic versus controlled processing (Posner & Snyder, 1975), we should either predict no effects on the N400 (conservative automaticity view) or we should find strong interference effects such as amplitude reduction and/ or temporal delay of the N400 (as an index of controlled processing). These effects might be even more severe for sentences than those observed at word level if we keep in mind that sentence reading is more complex. Thus, at short SOA, that is, at high overlap between sentence processing and additional task processing, we would expect a combination of latency postponement and amplitude attenuation of the N400 component. According to the more recent attentional sensitization view (Kiefer & Martens, 2010) one might alternatively assume that sentence processing strengthens the semantic pathway. Therefore we would expect at most a mild attenuation or delay of the N400, because the system flexibly enhances resources to process stimuli that are relevant for the task at hand.

Recently, another ERP component, the P600, has been discussed to—at least partially—reflect semantic processing. The P600 is a long-lasting positivity, sometimes beginning as early as 200 ms after stimulus onset, reaching its maximum between 600 and 800 ms. Initially, the P600 has been attributed exclusively to syntactic processing, because it was observed in the context of morphosyntactic violations for both written and acoustic input in different languages (Osterhout & Holcomb, 1992). More recently, the P600 has also been observed for pure semantic violations within sentences (e.g., Frisch, Schlesewsky, Saddy, & Alpermann, 2002; Hagoort, 2003; Martín-Loeches, Nigbur, Casado, Hohlfeld, & Sommer, 2006; Martín-Loeches et al., 2009). To account for such findings Kuperberg (2007) suggested that the P600 represents a combinatorial process that exploits both syntactic and semantic information for sentence interpretation. In addition to the N400 component in the present study also the P600 component was measured. It was of interest, whether effects on the N400 would be mirrored in the P600 or whether there would be dissociations. Because the P600 was not the focus of the present study, it will be reported only briefly.

## Experiment 1: Semantic Processing in a Single Task

To obtain baseline data and to test whether the Spanish sentences implemented in the PRP paradigm (see Exp. 2) would induce an N400 component, we first conducted a single task experiment that included all the stimuli for the PRP experiment, but only required acceptability judgments for the sentences; the additional tone was to be ignored here. Experiment 1 served as a check whether the materials and procedure would enable the recording of a N400 component and whether it would be modulated by the different temporal overlaps (SOAs) between target word and tones when no response to the tone was required.

### Method

#### Participants

Seventeen neurologically healthy, native Spanish-speaking university students (Faculty of Education) with normal or corrected-to-normal vision (15 women, age range 18 to 20 years) took part in the experiment in return for course credits. All were right-handed (Oldfield, 1971), with average handedness scores of +78, ranging from +28 to +100. Ethical guidelines were followed and participants signed an informed consent form.

#### Stimuli and Apparatus

The set of critical items consisted of 240 correct, acceptable Spanish sentences, of which 160 had been taken from Martín-Loeches et al. (2006). All sentences followed the same structure: determiner-noun-adjective-verb ([Det]-[N]-[Adj]-[V]). Expectancy for the adjectives in the acceptable sentences was assessed with a standard cloze probability procedure (cf. Kutas & Hillyard, 1984). The material was validated in two steps. First we performed a standard cloze probability procedure with 49 participants for the intact sentences. To do this with violating sentences does not make sense because violating sentences have almost zero close probability by definition. Mean cloze probability for the sentences was 4.6%, that is, for a given sentence nearly 5% of the 49 participants suggested the same penultimate adjective—followed by a verb. It was this adjective that was selected for the experimental sentence. In addition to the acceptable version of each sentence, an unacceptable version was created that contained a semantic violation due to an unacceptable combination of noun and adjective (e.g., El hielo frío desaparece. [The cold ice disappears.]; El hielo democrático desaparece. [The democratic ice disappears.]; see the Appendix for a list of examples). In order to ensure that the violating sentences are in fact unacceptable in a second step of validation we asked three additional persons to rate all experimental sentences for acceptability. Three raters were considered to be enough because there was very high agreement among them and across the violating sentences. In both acceptable and unacceptable versions of the sentences the critical words (the adjectives) were of comparable frequency (18.8 vs. 16.3 per million; *SE*s = 1.97 vs. 1.94), according to the “Lexico Informatizado del Espańol” (LEXESP; Sebastián, 2000). Furthermore, the number of letters for acceptable and semantically anomalous adjectives were very similar with *M*s = 7.8 and 7.7, respectively, *SE*s = 0.13 vs. 0.11.

Additionally, 100 filler sentences were included. Half of them contained four words, like the experimental sentences, but the adjective was omitted and an object or adverb was added (e.g., La grúa derriba muros. [The crane pulls down walls.]; El corazón late lento. [The heart beats slowly.]). The remaining fillers followed the structure of the experimental material, but verbs were transitive rather than intransitive and therefore required an object complement (e.g., El cocinero francés corta zanahorias. [The French cook cuts carrots.]). Half of the fillers were unacceptable sentences with semantic violations. Violations in the fillers occurred either in the verb or in the sentence’s final object. Filler sentences were included because pre-tests without fillers had shown that the tone served as a cue to the upcoming violation, occurring directly after the tone and always in the adjective of the experimental sentences. Hence participants did not have to deeply process the sentences and no N400 was induced. In the fillers the distance between tone and violation was more variable than in the experimental sentences (more words per sentence or the violation occurred in a different position and in a word category other than an adjective). In this way participants were forced to semantically process the sentences. All stimuli were presented in a white 20 pt font in the center of a black computer screen at a viewing distance of about 65 cm, yielding visual angles between 0.7ş and 1.3ş in height and 1.1ş to 6ş in width.

In addition to the visually displayed sentences, tones of 100 ms duration and 300 or 600 Hz were presented. For experimental sentences the tone always preceded the adjective at one of three possible SOAs. In case of filler sentences the tone occurred either before the subject, the predicate, or the sentence’s final object, being balanced over all filler sentences. Likewise, the SOA and, thus, the temporal overlap of tone and succeeding word was balanced. Manual responses to the sentences were recorded with two keys operated by the index fingers; no response to the tones was required. Stimulus presentation and recording of responses were controlled by ERTS^TM^ software (BeriSoft Company).

#### Procedure

**Figure 1.**
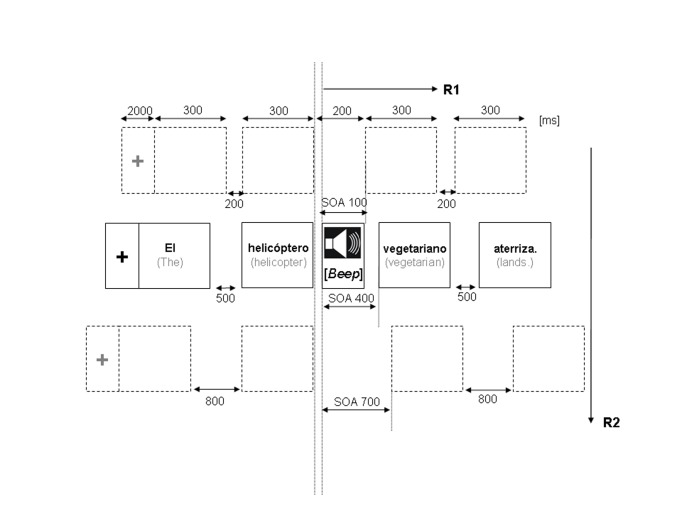
Chronometric depiction of an experimental trial (arbitrary scaling) showing a semantically unacceptable sentence. The single task experiment required manual responses only to the sentences’ acceptability. In the dual task experiment both foot responses to the tones and manual responses to the sentences had to be given.

The basic structure of an experimental trial is illustrated in Figure 1. A trial started with a fixation point in the center of the computer screen. After an interval of 2 s the experimental sentence was visually presented word by word, each word being shown for 300 ms. Tones were always presented 100 ms after the offset of the noun. Three SOAs between tone and adjective onset were used, 100, 400, or 700 ms. In order to present all words within a sentence at a regular pace the inter-stimulus-interval (ISI) between the words was adjusted to the SOA for a given sentence, yielding ISIs of 200, 500, and 800 ms, respectively (see also Figure 1).

From the pool of 240 acceptable sentences and their 240 unacceptable versions two sets of 240 experimental sentences each were created, containing 120 acceptable and 120 unacceptable versions. Within each set, no sentence was repeated and each sentence appeared only in one of its two versions (acceptable or unacceptable). SOA was randomly assigned to all the sentences within a set, with an equal amount of acceptable and unacceptable sentences within each SOA. Furthermore the assignments of high and low tones to each sentence were counterbalanced. All responses had to be given within 2.5 s after the sentence-final word, which was always shown with a period. A new experimental trial began either immediately after the response or when 2.7 s had elapsed since the offset of the final word.

Participants were seated in a dimly lit, sound attenuated chamber. By manually pressing the left or right key participants made acceptability judgments on each sentence. The response was to be given only after the end of the sentence. Participants were informed that they would hear high and low tones, which were, however, irrelevant to their task. Furthermore, they should keep their eyes fixed to the center of the screen.

Half of the participants responded to acceptable and unacceptable sentences with their right and left hand, respectively; for the others this assignment was reversed. The experiment consisted of 10 experimental blocks of 34 trials each (24 experimental and 10 filler trials). Preceding the experiment proper 24 practice trials were repeatedly presented until the participant responded correctly in all trials. Participants received visual feedback about the correctness of their response. After each block there was a short break.

#### EEG Recording and Data Analysis

The electroencephalogram (EEG) was recorded by means of 27 tin electrodes mounted within an electrode cap (ElectroCap International, ECI^TM^). Scalp locations according to the revised version of the 10/20 International System (American Electroencephalographic Society, 1991) were Fp1, Fp2, F7, F3, Fz, F4, F8, FC3, FC4, T7, C3, Cz, C4, T8, TP7, CP3, CP4, TP8, P7, P3, Pz, P4, P8, PO7, PO8, O1, O2, plus a left mastoid (M1) electrode. All electrodes were initially referenced to the right mastoid (M2). Bipolar horizontal and vertical electrooculograms (EOG) were recorded for artifact monitoring. Electrode impedances were kept below 5 k. ECI^TM^ electrode gel was used. The signals were recorded continuously with a band pass filter from 0.01 to 30 Hz and a sampling rate of 250 Hz.

The continuous EEG recording was initially segmented into 2200-ms epochs starting 200 ms before the onset of the adjectives in the experimental sentences. Artifacts were automatically rejected by eliminating EEG epochs exceeding a range of +200 V in any channel. Offline, ocular corrections for blinks, vertical, and horizontal eye movements were made using the method of Gratton, Coles, and Donchin (1983). Based on visual inspection epochs were eliminated that still presented artifacts. Epochs with incorrect judgments (i.e., acceptable sentences judged as unacceptable and incorrect sentences judged as acceptable) were also eliminated. After offline-rereferencing of the data to linked mastoids and the application of a 4-Hz low pass filter (24 db/ octave) to reduce residual noise, ERPs were averaged for each participant, electrode, and experimental condition. The 4-Hz low pass filter was chosen because the dual task situation made the data less stable than in other (single task) experiments. In the present data set (including Exp. 2) the 4-Hz filter yielded the most stable results, which are similar to what the authors found in previous dual task studies on the N400 with single words (Hohlfeld et al., 2004; Hohlfeld & Sommer, 2005, studies, in which a 7 Hz filter was applied). Furthermore the main power of the N400 component is around 3 Hz (Kutas & Van Petten, 1994)—still below the cutoff frequency of the filter used here.

All ERP waveforms were referred to a baseline, starting 100 ms prior to the target word, and analyzed in a time window of 1.5 s from target onset. This is the procedure employed in most other dual task studies with ERPs (e.g., Hohlfeld et al., 2004; Lien et al., 2008), to which the present data were to be compared.

**Figure 2.**
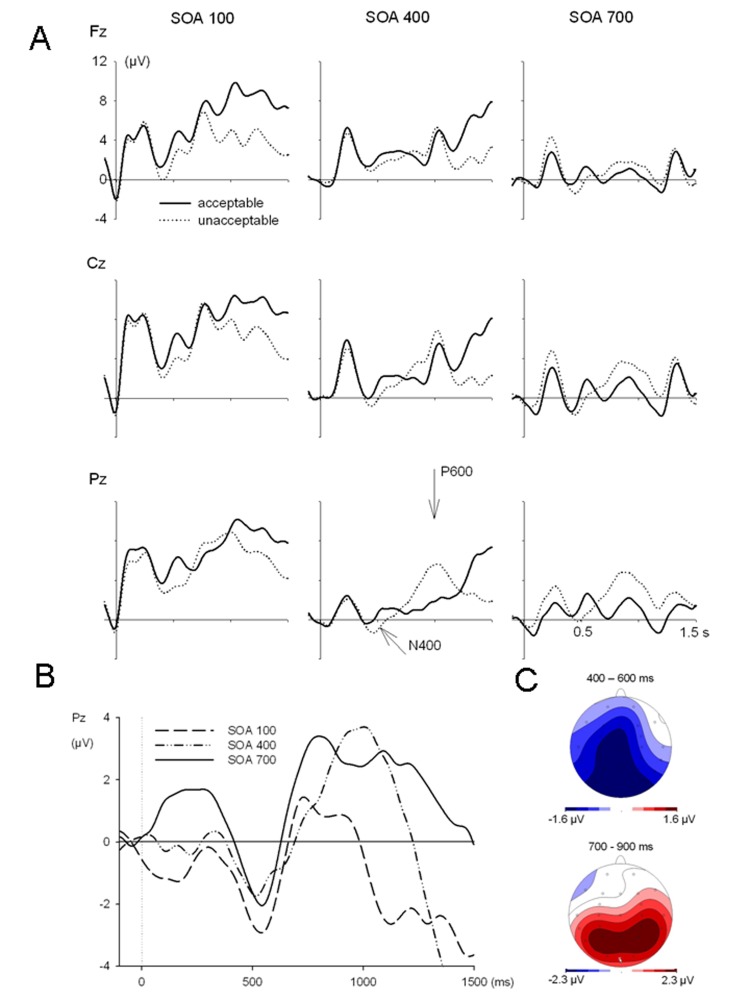
Event-related potentials from the Single Task Experiment 1, referred to a 100 ms prestimulus baseline. Panel A depicts ER P wave shapes at the Fz, Cz, and Pz electrode in response to acceptable and unacceptable targets at each SO A. Panel B superimposes the difference waves between ERPs to acceptable and unacceptable target words. Panel C shows the topography of difference wave amplitudes between 400 to 600 ms as well as between 700 to 900 ms after target onset; data were collapsed across the SOA conditions (N400 and P600 components, respectively).

The dual task paradigm provides a particular challenge to ERP methodology, because each stimulus and response is related to a complex set of brain waves that overlap in different ways depending on the SOA. This makes it difficult to disentangle effects in the composite waveforms. However, it is possible to isolate ERP components that relate to only one experimental factor by eliminating invariant overlapping activity with a subtraction procedure. This is in accordance with the assumption of Nunez (1981) that electric fields of several sources combine linearly without interacting. The logic of subtraction works if the overlapping activity is the same in the two subtracted conditions. Examples for this approach to dual task designs are studies by Luck (1998), Osman and Moore (1993), Sommer, Leuthold, and Schubert (2001), and Hohlfeld et al. (2004). This logic was also applied in the present study in order to isolate the N400 as well as the P600 component. Both components were obtained by subtracting ERPs to acceptable targets from those to unacceptable targets within each SOA. In this way we were able to isolate the electrophysiological response to word incongruity for conditions of different temporal overlap. Mean amplitude measures were calculated in two 200-ms time windows between 100 to 300 and 400 to 600 ms relative to the onsets of the critical words. The second window was chosen around the peak of the N400 component; the first window served to test a conspicuous effect seen in the wave shapes (cf. Fig. 2B). In addition, six consecutive 100-ms time windows between 650 and 1150 ms covered the typical time range of the P600 effects.

ERP amplitude measures were submitted to ANOVAs, with repeated measures on factors SOA (3 levels), acceptability (2 levels), and electrode site (27 levels) as independent variables. Error rates were subjected to ANOVAs, with repeated measurements on SOA and acceptability. If appropriate, degrees of freedom were corrected according to Huynh and Feldt (1976). Due to the fact that responses were only given after the sentence, reaction times for the sentences cannot be considered to represent processing times for the critical words and were therefore disregarded.

### Results

#### Performance

Error rates were higher for acceptable than unacceptable sentences (*M* = 20.4 vs. 15.0 %, *SE* = 1.89 vs. 2.39), *F*(1, 16) = 5.65, *p* < .05. For factor SOA there was neither a significant main effect nor an interaction with any of the other factors.

#### ERP Data

Figure 2A shows the grand mean ERPs for acceptable and unacceptable target words at selected electrodes (Fz, Cz, and Pz) in the three SOA conditions. Difference waves at each SOA are depicted in Figure 2B for electrode Pz. To visualize the topography of N400, data was collapsed across the three SOAs during the time window 400-600 ms. Figure 2C displays the scalp topography of a widely distributed N400 effect (inacceptable minus acceptable words) along the midline and at centro-parietal electrodes.

ANOVA (Table 1) indicated no effects of acceptability in the time window between 100 and 300 ms. However, acceptability effects were statistically confirmed for the N400 time window, that is, between 400 to 600 ms. In a first step of analysis the interaction Acceptability × Electrode was significant, *F*(26, 416) = 1.92, *p* = .005, however after Huynh and Feldt correction the effect failed significance. In the N400 time window the acceptability effect was not significantly modulated by SOA. A post-hoc test at Cz and Pz electrodes was performed, which yielded significant main effects of SOA, *F*(2, 32) = 10.5, *p* = .000, and acceptability, *F*(1, 16) = 18.7, *p* = .001, confirming the presence of an N400 at these typical sites. There was no interaction of SOA and acceptability. Acceptability effects were statistically confirmed also between 750 to 1050 ms (cf. Table 1), representing the P600 component, which showed the usual scalp distributions and latencies (see Fig. 2B and 2C, bottom). Interestingly, there were effects of SOA on later time segments of the P600, starting at 950 ms.

**Table 1.** *F* Values and Significance Levels From the ANOVAs of ERP Amplitudes in Experiments 1 and 2

	Start of Time Segment [ms]								
	*d*_f_	100^a^	400	650	750	850	950	1050	1150
Experiment 1									
SOA	2, 32	7,43**	5,07*	18,17***	27,2***	18,24***	13,77***	17,2***	17,98***
SOA*Electr	52, 832	4,3*	2,77*	7,57***	7,17***	4,92***	4,21**	3,6**	4,74**
Acc	1, 16	--	11,48**	--	6,58*	7,78*	4,22*	--	--
Acc*Electr	26, 416	--	--	--	4,95**	8,81***	8,02***	7,79***	4,5**
SOA*Acc	2, 32	--	--	--	--	--	7,46**	16,03***	5,9**
SOA*Acc*Electr	52, 832	--	--	--	--	--	2,3*	2,81**	2,08*
									
Experiment 2									
SOA	2, 38		5,46*	28,63***	28,01***	19,79***	16,8***	17,99***	39,09***
SOA*Electr	52, 988		19,99***	18,57***	17,46***	17,36***	10,45***	11,45***	10,27***
Acc	1, 19		22,05***	--	--	4,4*	(.07)	--	--
Acc*Electr	26, 494		3,38*	--	--	3,96**	5,56**	5,07**	6,13***
SOA*Acc	2, 38		3,57*	--	--	(.09)	--	5,76**	8,17**
SOA*Acc*Electr	52, 988		--	--	--	--	--	--	2,21*

*Note*. *p < .05. **p < .01. ***p < .001. ^a^Segments starting at 100 and 400 ms were of 200 ms duration; those from 650 ms onwards were of 100 ms duration. Acc: Acceptability, Electr: Electrode

### Discussion

As expected, this single task experiment yielded an N400 component to the target words in unacceptable relative to acceptable sentences. The N400 component showed a broad scalp distribution with a maximum at centro-parietal electrodes, and with a typical latency. No SOA effects on the N400 amplitude occurred, indicating that the tone as such and the SOA variation of the intervals between words did not affect the N400, providing a good baseline for the dual-task Experiment 2. At the behavioral level the participant’s acceptability judgments disagreed more often with the experimenters’ definition for acceptable than for unacceptable sentences, but this was independent of SOA. The general error rate was quite high, around 20%. This implies that the acceptability task was relatively difficult. As shown by Martens & Kiefer (2009) unconscious automatic semantic processing is modulated by the difficulty of a primary task. When the primary task was hard (decide whether a masked word begins or ends with an open shaped or closed letter, as mentioned above) the priming effect was absent in a lexical decision task, compared to an easy primary task. Thus, a difficulty effect of the actual semantic task on the semantic processing stream cannot be ruled out in the present experiment. Importantly, difficulty did not vary across SOAs (otherwise we would have found a significant interaction between Acceptability and SOA in the error rates of the acceptability task). Therefore difficulty of the semantic task is not considered to be a confound with the dual task manipulation in Experiment 2.

In contrast to the N400, the P600 component seemed to be sensitive to the overlapping tone stimulus as reflected by an amplitude reduction of the P600 with decreasing SOA. The effects of SOA on the P600 could also be due to limited processing time at short intervals between words in a sentence (Ainsworth-Darnell, Shulman, & Boland, 1998; Kotz, von Cramon, & Friederici, 2005).

## Experiment 2: Semantic Processing in a Dual Task

The aim of this experiment was to determine whether an additional task would interfere with semantic processing during sentence reading and to investigate the pattern of a possible interference effect. The same stimulation procedure was used as in the single-task Experiment 1, but now, in addition to the sentence acceptability task, the previously irrelevant tones required choice responses. The different intervals between tone and target word (adjective), together with the instructed priority of the tone discrimination task aimed at producing a PRP paradigm with variable SOAs (see Fig. 1). The SOA variation manipulated the temporal overlap between the processes required by the choice response to the tone and the processing of semantically acceptable and unacceptable adjectives in the sentences.

### Method

#### Participants

The experiment involved 20 neurologically healthy participants (16 women) with normal or corrected-to-normal vision; all of them were native Spanish speaking university students (Faculty of Education) and received course credits for participation. Mean age was 20.05 years (range 18-26 years); 18 participants were right-handed, two were left-handed (with average handedness scores of +64, ranging from -100 to +100). Ethical guidelines were followed and participants signed an informed consent form.

#### Materials

The material was mostly the same as in the single-task Experiment 1; two acceptable adjectives were replaced because they had repeatedly caused incorrect responses. Mean values of cloze probability, number of letters, and word frequency of acceptable and unacceptable adjectives were not affected by these changes.

#### Procedure

Apart from the additional task, the procedure was the same as in Experiment 1. Accordingly, manual acceptability decisions had to be made to the visually presented sentences; but now, also the tones were relevant, requiring choice responses. High- and low-pitched tones had to be responded to with the left or right foot on keys, embedded in a footrest. The keys were pressed with the big toes, shoes being taken off.

The assignment of tone stimuli to response feet was counterbalanced across participants. Choice responses to the tones were to be executed with priority over the sentence acceptability decisions. Temporal overlap (SOAs) of the tones with the critical words in the sentences was again varied in three levels (100, 400, 700 ms).

As in Experiment 1 ten blocks of 34 trials were presented. Sixteen versions of the experiment were generated balancing acceptable and unacceptable versions of the target words, mapping of hand and acceptability, and stimulus-response assignment of the tone task. High and low-pitched tones occurred equiprobably in each condition combination and independent of the acceptability condition. To familiarize participants with the dual task requirements, three practice blocks preceded the experiment proper, which were repeated until participants responded correctly. Visual feedback was given during practice. Foot and hand responses were first practiced in single task blocks and then in combination in one dual task block. For practice trials other language stimuli were used than in the main experiment.

#### Data Analysis

Data analysis of the behavioral and ERP responses to the visually presented adjectives was conducted as in the single-task Experiment 1, with factors SOA (now indicative of the effects of the additional task), acceptability (indicator of semantic processing), and electrode. Furthermore, also performance (reaction times and error rates) in the additional tone discrimination task was analyzed with repeated measures of SOA and acceptability.

### Results

#### Additional Task Performance

Responses in the tone discrimination task at SOA 100 were somewhat more error-prone than at SOAs 400 and 700 (*M*s = 4.18, 2.62, and 2.93%, *SE*s = 1.10, 0.66, and 0.73, respectively), *F*(2, 38) = 3.22, *p* =.05. Tone discrimination accuracy was neither affected by sentence acceptability as main effect nor in interaction with SOA (*F*s < 1). Reaction times in the tone discrimination task were neither significantly affected by SOA, *F* = 1.48 (M = 709.19 ms, SE = 31.19 over all conditions) nor by sentence acceptability, nor by an interaction between sentence acceptability and SOA (*F*s = 2.59 and < 1.00, respectively).

#### Sentence Acceptability Task Performance

Correctness of sentence acceptability judgments was significantly affected by acceptability. Acceptable sentences yielded fewer erroneous responses than unacceptable ones (*M*s = 16.29 vs. 20.70%, *SE*s = 1.30 vs. 1.72, respectively), *F*(1, 19) = 8.69, *p* < .01. In contrast, to Experiment 1, there was also an effect of SOA: Error rates decreased from short to long SOA (*M*s = 24.25, 16.43, 14.81%, *SE*s = 1.82, 1.77, 1.39), *F*(2, 38) = 16.56, *p* < .001. Furthermore SOA and acceptability interacted, *F*(2, 38) = 4.74, *p* < .05 (see Table 2 for further details). Post-hoc, Bonferroni-corrected, pair-wise comparisons of acceptable and unacceptable sentences at each SOA revealed a significant difference only for the short SOA, *F*(1, 19) = 9.83, *p* < .01.

**Table 2.** Error Rates for the Sentence Acceptability Task of Experiment 2

Target	SOA 100	SOA 400	SOA 700
Acceptable	19.37	15.00	14.50
	(1.99)	(1.76)	(1.36)
Unacceptable	29.12	17.87	15.12
	(2.74)	(2.23)	(1.87)

*Note*. Mean values in percent, standard errors in parentheses.

#### ERP Data

**Figure 3.**
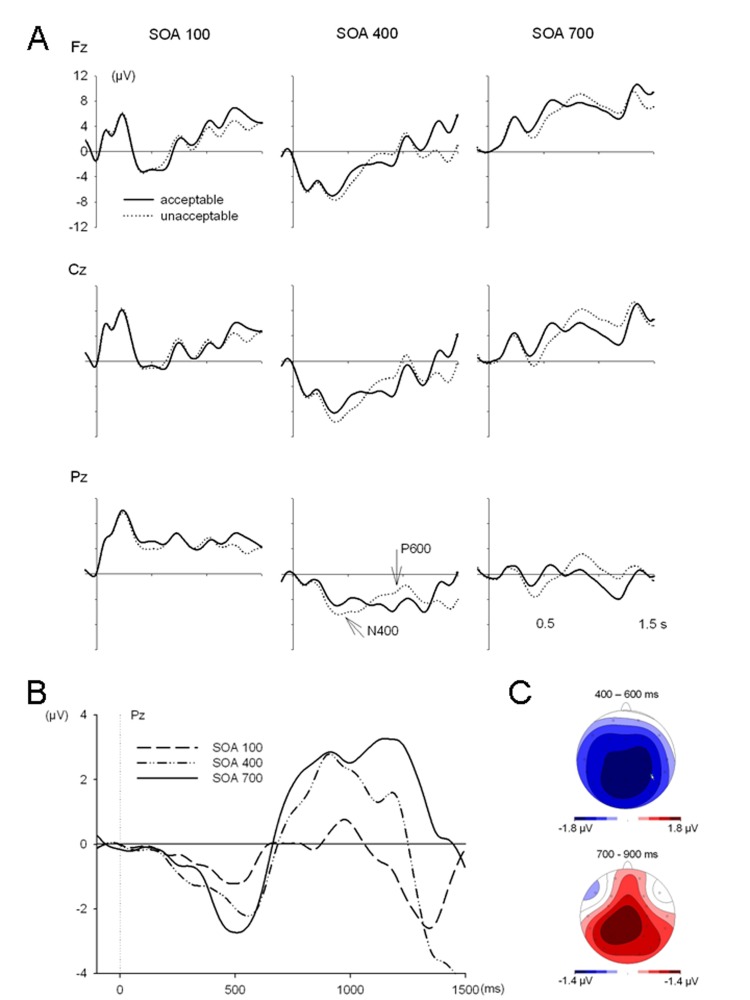
Event-related potentials from the Dual Task Experiment 2, referred to a 100 ms prestimulus baseline. Panel A depicts ERP wave shapes at the Fz, Cz and Pz electrode in response to acceptable and unacceptable target words at each SOA. Panel B superimposes the difference waves between ERPs to acceptable and unacceptable target words. Panel C shows the topographies of difference wave amplitudes between 400 to 600 ms as well as between 700 to 900 ms after target onset (N400 and P600 components, respectively). Data were collapsed across the SOA conditions.

Figure 3A depicts grand average ERPs evoked by acceptable and unacceptable target adjectives for the three SOA conditions at selected electrodes (Fz, Cz, and Pz). Again, as in the single-task Experiment 1, differences in amplitude between acceptable and unacceptable target nouns were evident in the N400 time window.

Difference waves of the grand averages are depicted in Figure 3B, superimposed for the three SOA conditions at the Pz electrode. The amplitudes of these difference waves between 400 and 600 ms seem to be modulated by SOA, being smallest at SOA 100. Figure 3C depicts the scalp topography of the N400 component (time window 400-600 ms), which again is a widely distributed negativity along the midline and at centro-parietal electrodes.

The patterns we observed in this experiment for the acceptability effects were long-duration difference waves. Consequently, it was not feasible to measure peak latencies or peak amplitudes. Instead, the quantitative analysis was restricted to average amplitude measures between 400 and 600 ms. As can be seen in Table 1 there was a significant effect of acceptability between 400 and 600 ms, which significantly interacted with the factors SOA and Electrode. In a further step the factor Acceptability was analyzed within each SOA. At SOA 100 it failed significance, *F*(1, 19) = .49, *p* > .05, but at SOA 400 and 700 this factor was significant (SOA 400: *F* = 4.86, *p* < .05, SOA 700: *F* = 18.83, *p* < .001). Acceptability did not interact with factor Electrode although at SOA 700 there was a trend for such an interaction (*p* = .09). In addition, an analysis including selected electrodes (F3, Fz, F4, Fc3, Fc4, C3, Cz, C4, Cp3, Cp4, P3, Pz, P4, O1 and O2) was run. This yielded significant main effects of SOA, *F*(2, 38) = 4.9, *p* = .016, and acceptability, *F*(1, 19) = 21.35, *p* = .000, as well as a strong trend for an interaction between these factors, *F*(2, 38) = 3.1, *p* = .057. Tests at single electrodes (Cz or Pz only) did not yield significant results, possibly because of the wide distribution of the N400.

Differences in amplitude between acceptable and unacceptable target nouns were again evident in the P600 time windows. This time, the P600 seemed to be affected by SOA, with a reduction in amplitude (particularly at short SOA) already in an earlier time window (between 850 and 950 ms, cf. Table 1) as compared to Experiment 1. However, there was only a trend for an interaction with SOA (*p* = .09). Excluding the factor electrode and running analyses only for the Pz electrode, where this component was largest, this interaction was significant, *F*(2, 38) = 3.96, *p* < .05. As in Experiment 1, effects of acceptability were found in the later P600 time windows, between 1050 and 1250 ms.

### Discussion

It was the central question of this dual task experiment whether semantic processing of written words within sentences would be attenuated by an additional non-linguistic task. Reaction times in the additional high-priority task were not affected by the SOA manipulation. In contrast, the additional tone task caused an increase of error rates in the sentence acceptability performance when temporal overlap between the tasks was high (SOA 100). On the ERP level, responses to the additional tone task at short SOA caused amplitude attenuations of the N400 component to the critical words. Although the semantic task per se was quite difficult, as indicated by high error rates in Experiment 1, it was not strongly postponed under conditions of high task overlap. In the following the pattern of results from both experiments shall be discussed in the context of the controversy of automatic versus controlled semantic processing.

## General Discussion

The general aim of the present study was to investigate the effects of an additional task on indicators of semantic processing at sentence level. Main indicator of semantic processing was the N400 component of the ERP. According to the traditional automaticity view (Posner & Snyder, 1975; Schneider & Shiffrin, 1977) semantic processing would qualify as an automatic process only, if the N400 would be unaffected by high temporal overlap with an additional task. In contrast, if semantic processing were a non-automatic or controlled process, latency postponement or amplitude attenuation of the N400 component would be expected under conditions of high overlap. However, the more recent attentional sensitization model (Kiefer & Martens, 2010) predicts only mild attenuations of the N400 in a sentence context due to strengthening of the semantic pathway or priming from one word to the other (as a type of cuing).

What we found in the present study were amplitude reductions that might be considered as rather mild effects, in the sense that the N400 component was not drastically delayed. In contrast, in their dual task study with word pairs Hohlfeld et al. (2004) had reported a delay of the N400 peak latency of about 270 ms. Interestingly, although processing of words at sentence level is more complex than of isolated words and the present acceptability task was quite difficult, the observed effects are—if anything—less severe than those observed at word (pair) level. Hence, processing at sentence level seems to support semantic processing.

Additionally, in Experiments 1 and 2 (single and dual task, respectively) we observed a P600 component. Its later segments (950 ms-1250 ms) were already modulated by the SOA in the single task of Experiment 1. In the dual task of Experiment 2, we observed interference effects in both earlier (850 ms-950 ms) as well as later parts (1050 ms-1250 ms). The mild effects on the P600 observed in Experiment 2 mirror the mild effects on the N400. Thus, we suggest that integrative processing as well as semantic processing were relatively stable during sentence processing.

The observed patterns of results are difficult to explain in the context of a traditional understanding of automaticity but are in line with the attentional sensitization model. We assume that the semantic processing pathway at sentence level is strengthened by continuously on-going priming from one word to the other. Kiefer and Brendel (2006) demonstrated such enhancing effects of cueing in a masked priming paradigm. Additionally, in the present paradigm, where the target word was not the last word in the sentence, the upcoming final word might have speeded up processing of the preceding word and thus might not have “allowed” any delay, especially at short SOA, where the last word followed the target word by only 200 ms.

The PRP paradigm is usually employed to investigate whether a processing stream requires a central process or depends on central attention. From interference effects at high overlap between tasks (short SOA) the conclusion is drawn that the affected process is a controlled, non-automatic process that requires central attention or occupies a central processing bottleneck. As far as semantic processing is concerned interference effects in terms of temporal delays have been reported. At word level Lien et al. (2008) and Hohlfeld et al. (2004) found postponements of N400 peak latencies at high temporal overlap with an additional task. These latency shifts of the N400 component were interpreted in terms of time-sharing (Pashler & Johnston, 1989) between the additional and the verbal task. It was suggested that semantic processing is temporarily halted by a central processing bottleneck, as long as this bottleneck is occupied by the additional task. As only central processes are affected by this bottleneck semantic processing was seen as a central and thus as a controlled, non-automatic process.

Apart from latency delays also amplitude reductions of the N400 have been observed in dual task studies (Hohlfeld & Sommer, 2005, as well as Rabovsky, Álvarez, Hohlfeld, & Sommer, 2008; Vachon & Jolicoeur, 2011). The observed modulations of the N400 by the additional task were accounted for by shifts of central attention. With respect to the question whether semantic processing might be automatic or controlled, the dependency of the N400 and thus of semantic processing on central attention was again taken as an index of controlled processing. According to the working memory account of Baddeley (1986), mental resources—that is, central attention, and its allocation to specific aspects of the task at hand are administered by the central executive. This administration of mental resources is believed to be controlled and non-automatic. Dual-task interference effects have been explained in terms of shared central attention by other types of cognitive operations (cf. Navon & Miller, 2002; Tombu & Jolicoeur, 2003). A similar interpretation based on the depletion of processing resources for one of the tasks was used to explain amplitude reductions of the P300 component (Luck, 1998).

The amplitude reductions in the N400 in the present study are difficult to integrate into a model that assumes a mere postponement of processing stages as suggested by the bottleneck account (cf. Hohlfeld & Sommer, 2005, for a similar finding and argumentation). Also, resource models of dual task processing cannot account for the effects. If resources were shifted from the primary to the secondary task to maintain stable semantic processing, one would expect effects of SOA on error rates and reaction times in the primary task. However, these were not found and the actual mechanisms of interference are still a matter of debate. Vachon and Jolicoeur (2012) measured the N400 component in a PRP paradigm under conditions of task switching (Task 1 number discrimination, Task 2 semantic task) or no switching. Attenuations of the N400 component were observed only when a switch from a perceptual to a semantic or between different semantic tasks occurred. Interestingly, when Tasks 1 and 2 were the same, the N400 was not attenuated at short intervals. This finding was interpreted in line with the attentional sensitization model. Semantic processing is automatic to the extent that it can survive multitasking. Thus, it was concluded that semantic processing is not dependent on central attention during response selection or during decision making. When the cognitive requirements were the same in both tasks, no interference occurred. The authors suggested that the N400 and consequently semantic processing is not susceptible to the processing bottleneck. However, semantic processing is susceptible to reconfiguration of the task set.

Thus, the amplitude reductions of the N400 observed in the present study could be explained by such reconfigurations of the task set by SOA. Additionally, mechanisms are assumed that strengthen the semantic processing stream. To enhance performance the cognitive system might be more flexible in dual task situations than previously believed. Such a notion of flexibility is supported by findings from Lien et al. (2008, Exp. 4), in which target words preceded the additional stimulus and, as in the other experiments, participants were instructed to primarily respond to the additional stimulus. What the authors found was the same attenuation of the N400 as when the verbal target succeeded the additional stimulus, indicating that attention was strategically shifted to the stimulus to be processed first and not to the stimulus that appeared first.

Although we have to concede that the signal-to-noise ratio of the present studies was not optimal, the effects observed are in line with others and are theoretically plausible. Nevertheless further studies are needed to confirm and extend the present findings, which—to our knowledge—are the first at sentence level. In addition future research on semantic processing at sentence level might address the question, how the nature of the embedding sentences, for example their syntactic complexity or semantic constraints, will modulate the observed protective effect in a dual task situation.

In sum, the findings from the present study contribute to the debate on the nature of semantic processing during reading and extend it to sentence level processing. Semantic processing was neither left completely intact nor strongly postponed by the additional task. It is suggested that the observed amplitude reductions of the N400 component were caused by a reconfiguration of the task set by the overlapping task, whereas the absence of postponement reflects a protective effect of the sentence context. This supports the idea of a flexible cognitive system that is able to strengthen task-relevant pathways. The model of attentional sensitization has integrated this notion of flexibility and suggests that also automatic processes can be modulated according to task demands. In this sense, the present findings indicate that also semantic processing at sentence level is an automatic yet flexible process.

## APPENDIX

List of 16 example sentences (out of a set of 240) with semantically appropriate and inappropriate target words. Corresponding English non-literal translations are given in parantheses.

La profesora rigurosa/ fluida explica.

(The strict/ liquid teacher explains.)

El sastre diligente/ aromático cose.

(The diligent/ aromatic tailor sews.)

La biblioteca municipal/ realista abre.

(The local/ realistic library opens.)

Las pelotas moradas/ listas botan.

(The violet/ clever balls jump.)

El helicóptero sanitario/ vegetariano aterriza.

(The rescue/ vegetarian helicopter lands.)

La comida mexicana/ próspera pica.

(The Mexican/ wealthy dish is spicy hot.)

El vendaval incesante/ cansado reincide.

(The incessant/ tired storm recedes.)

La hermana querida/ jugosa llora.

(The beloved/ juicy sister is crying.)

El jardinero laborioso/ lateral planta.

(The hardworking/ lateral gardener is planting.)

La policía nacional/ crujiente dispara.

(The national/ crispy police is shooting.)

Las ruedas pinchadas/ educadas chillan.

(The punctured/ educated wheels squeal.)

El hombre delicado/ nuboso fuma.

(The delicate/ cloudy man is smoking.)

El vagabundo miserable/ venenoso pide.

(The miserable/ poisoning vagabond is begging.)

La cama blanda/ gustosa cabe.

(The soft/ tasty bed fits.)

El ruido molesto/ mofletudo reduce.

(The disturbing/ chubbycheeked noise is diminishing.)

La chaqueta ligera/ triangular abriga.

(The light/ triangular jacket protects.)
